# Mining Genomes of Three Marine Sponge-Associated Actinobacterial Isolates for Secondary Metabolism

**DOI:** 10.1128/genomeA.01106-15

**Published:** 2015-10-01

**Authors:** Hannes Horn, Ute Hentschel, Usama Ramadan Abdelmohsen

**Affiliations:** a Department of Botany II, Julius-von-Sachs Institute for Biological Sciences, University of Würzburg, Würzburg, Germany; b Department of Marine Microbiology, GEOMAR Helmholtz Centre for Ocean Research, RD3 Marine Microbiology and Christian-Albrechts University of Kiel, Kiel, Germany

## Abstract

Here, we report the draft genome sequences of three actinobacterial isolates, *Micromonospora* sp. RV43, *Rubrobacter* sp. RV113, and *Nocardiopsis* sp. RV163 that had previously been isolated from Mediterranean sponges. The draft genomes were analyzed for the presence of gene clusters indicative of secondary metabolism using antiSMASH 3.0 and NapDos pipelines. Our findings demonstrated the chemical richness of sponge-associated actinomycetes and the efficacy of genome mining in exploring the genomic potential of sponge-derived actinomycetes.

## GENOME ANNOUNCEMENT

Actinomycetes are known for their unprecedented ability to produce novel lead compounds of clinical and pharmaceutical importance (1–4). Among the many actinobacterial genera, *Streptomyces*, *Micromonospora*, *Nocardiopsis*, and *Rhodococcus* are the most prolific producers of secondary metabolites, which display broad chemical diversity and diverse pharmaceutically and medically relevant bioactivities (5–8). Recent genomic sequencing data have revealed the presence of a plethora of putative biosynthetic gene clusters on the genomes of actinomycetes encoding for secondary metabolites that are not observed under standard fermentation conditions (9–13). In the present study, draft genomes of three actinobacterial isolates, *Micromonospora* sp. RV43, *Rubrobacter* sp. RV113, and *Nocardiopsis* sp. RV163 that had previously been cultivated from the Mediterranean sponges *Aplysina aerophoba* (RV43 and RV113) and *Dysidea avara* (RV163) (14), were established.

The genomic DNA of the isolates was extracted from 5-day-old ISP2 cultures. Paired-end, 2 × 250-bp libraries were prepared with the Nextera XT kit (Illumina, Inc.). Sequencing was performed on an Illumina MiSeq device. A total of 5,900,702 raw reads were produced for *Micromonospora* sp. RV43, 2,206,732 raw reads for *Rubrobacter* sp. RV113 and 4,851,980 raw reads were delivered for *Nocardiopsis* sp. RV163. Reads were adapter clipped, quality trimmed and length filtered (15). Initial contigs were generated using SPAdes (16) and only contigs ≥1000 bp were maintained. A further clean-up of contigs was performed using G+C-content, coverage, and taxonomic assignments (17). For *ab initio* gene prediction, prodigal was applied (18) and functional annotation of the predicted protein sequences was performed with the RAST webserver (19). Secondary metabolite gene clusters and possible encoded compounds were predicted with antiSMASH (20) and NapDos (21).

A number of 101 (RV43), 33 (RV113), and 82 (RV163) secondary metabolite gene clusters were detected with antiSMASH. For strain RV43, 5 terpene clusters, 4 type 1 PKS clusters, 2 lantipeptides, 1 type 2 PKS cluster, 1 siderophore, 1 NRPS cluster, and 1 bacteriocin were found. For strain RV113, 3 terpene clusters, 1 fatty acid, and 1 mixed type 3 PKS-fatty acid cluster were found. The draft genome sequence of strain RV163 showed homologies to 7 NRPS clusters, 4 terpene gene clusters, 2 type 1 PKS clusters, 2 ectoines, 2 bacteriocins, 1 phenanzine, 1 butyrolacetone, 1 type 2 PKS, and 1 siderophore.

For *Micromonospora* sp. RV43, NaPDoS predicted the presence of gene clusters encoding for compounds such as leinamycin, kirromycin, aclacinomycin, and tetronomycin. For *Nocardiopsis* sp. RV163, compounds such as alnumycin, avermectin, and neocarzinostatin were predicted. For *Rubrobacter* sp. RV113, only gene clusters encoding for fatty acids synthesis were found. These results highlight the genomic potential of at least two of three inspected isolates for natural products discovery.

### Nucleotide sequence accession numbers.

This whole-genome shotgun project was deposited in DDBJ/ENA/GenBank under the accession numbers LEKG00000000, LEKH00000000, and LEKI00000000. The versions described in this paper are the first versions LEKG01000000, LEKG01000000, and LEKH01000000.

## References

[1] Abdelmohsen UR, Bayer K, Hentschel U 2014 Diversity, abundance and natural products of marine sponge-associated actinomycetes. Nat Prod Rep 31:381–399. doi:10.1039/C3NP70111E. 24496105

[2] Li JW, Vederas JC 2009 Drug discovery and natural products: end of an era or an endless frontier? Science 325:161–165. doi:10.1126/science.1168243. 19589993

[3] Eltamany EE, Abdelmohsen UR, Ibrahim AK, Hassanean HA, Hentschel U, Ahmed SA 2014 New antibacterial xanthone from the marine sponge-derived *Micrococcus* sp. EG45. Bioorg Med Chem Lett 24:4939–4942. doi:10.1016/j.bmcl.2014.09.040. 25283555

[4] Grkovic T, Abdelmohsen UR, Othman EM, Stopper H, Edrada-Ebel R, Hentschel U, Quinn RJ 2014 Two new antioxidant actinosporin analogues from the calcium alginate beads culture of sponge-associated *Actinokineospora* sp. strain EG49. Bioorg Med Chem Lett 24:5089–5092. doi:10.1016/j.bmcl.2014.08.068. 25266784

[5] Reimer A, Blohm A, Quack T, Grevelding CG, Kozjak-Pavlovic V, Rudel T, Hentschel U, Abdelmohsen UR 20 5 2015 Inhibitory activities of the marine streptomycete-derived compound SF2446A2 against *Chlamydia trachomatis* and *Schistosoma mansoni*. J Antibiot. doi:10.1038/ja.2015.54. 25990954

[6] Dashti Y, Grkovic T, Abdelmohsen UR, Hentschel U, Quinn RJ 2014 Production of induced secondary metabolites by a co-culture of sponge-associated actinomycetes, *Actinokineospora* sp. EG49 and *Nocardiopsis* sp. RV163. Mar Drugs 12:3046–3059. doi:10.3390/md12053046. 24857962 PMC4052330

[7] Abdelmohsen UR, Yang C, Horn H, Hajjar D, Ravasi T, Hentschel U 2014 Actinomycetes from Red Sea sponges: sources for chemical and phylogenetic diversity. Mar Drugs 12:2771–2789. doi:10.3390/md12052771. 24824024 PMC4052315

[8] Abdelmohsen UR, Szesny M, Othman EM, Schirmeister T, Grond S, Stopper H, Hentschel U 2012 Antioxidant and anti-protease activities of diazepinomicin from the sponge-associated *Micromonospora* strain RV115. Mar Drugs 10:2208–2221. doi:10.3390/md10102208. 23170078 PMC3497017

[9] Harjes J, Ryu T, Abdelmohsen UR, Moitinho-Silva L, Horn H, Ravasi T, Hentschel U 2014 Draft genome sequence of the antitrypanosomally active sponge-associated bacterium *Actinokineospora* sp. strain EG49. Genome Announc 2(2):e00160-14. doi:10.1128/genomeA.00160-14. 24604655 PMC3945511

[10] Abdelmohsen UR, Grkovic T, Balasubramanian S, Kamel MS, Quinn RJ, Hentschel U 2015 Elicitation of secondary metabolism in actinomycetes. Biotechnol Adv 33:798–811. doi:10.1016/j.biotechadv.2015.06.003. 26087412

[11] Challis GL 2008 Mining microbial genomes for new natural products and biosynthetic pathways. Microbiology 154:1555–1569. doi:10.1099/mic.0.2008/018523-0. 18524911

[12] Jensen PR, Chavarria KL, Fenical W, Moore BS, Ziemert N 2014 Challenges and triumphs to genomics-based natural product discovery. J Ind Microbiol Biotechnol 41:203–209. doi:10.1007/s10295-013-1353-8. 24104399 PMC3946964

[13] Nett M, Ikeda H, Moore BS 2009 Genomic basis for natural product biosynthetic diversity in the actinomycetes. Nat Prod Rep 26:1362–1384. doi:10.1039/b817069j. 19844637 PMC3063060

[14] Abdelmohsen UR, Pimentel-Elardo SM, Hanora A, Radwan M, Abou-El-Ela SH, Ahmed S, Hentschel U 2010 Isolation, phylogenetic analysis and anti-infective activity screening of marine sponge-associated actinomycetes. Mar Drugs 8:399–412. doi:10.3390/md8030399. 20411105 PMC2857355

[15] Bolger AM, Lohse M, Usadel B 2014 Trimmomatic: a flexible trimmer for Illumina sequence data. Bioinformatics 30:2114–2120. doi:10.1093/bioinformatics/btu170. 24695404 PMC4103590

[16] Bankevich A, Nurk S, Antipov D, Gurevich AA, Dvorkin M, Kulikov AS, Lesin VM, Nikolenko SI, Pham S, Prjibelski AD, Pyshkin AV, Sirotkin AV, Vyahhi N, Tesler G, Alekseyev MA, Pevzner PA 2012 SPAdes: a new genome assembly algorithm and its applications to single-cell sequencing. J Comput Biol 19:455–477. doi:10.1089/cmb.2012.0021. 22506599 PMC3342519

[17] Camacho C, Coulouris G, Avagyan V, Ma N, Papadopoulos J, Bealer K, Madden TL 2009 BLAST+: architecture and applications. BMC Bioinformatics 10:421. doi:10.1186/1471-2105-10-421. 20003500 PMC2803857

[18] Hyatt D, Chen GL, Locascio PF, Land ML, Larimer FW, Hauser LJ 2010 Prodigal: prokaryotic gene recognition and translation initiation site identification. BMC Bioinformatics 11:119. doi:10.1186/1471-2105-11-119. 20211023 PMC2848648

[19] Aziz RK, Bartels D, Best AA, DeJongh M, Disz T, Edwards RA, Formsma K, Gerdes S, Glass EM, Kubal M, Meyer F, Olsen GJ, Olson R, Osterman AL, Overbeek RA, McNeil LK, Paarmann D, Paczian T, Parrello B, Pusch GD, Reich C, Stevens R, Vassieva O, Vonstein V, Wilke A, Zagnitko O 2008 The RAST server: Rapid Annotations using Subsystems Technology. BMC Genomics 9:75. doi:10.1186/1471-2164-9-75. 18261238 PMC2265698

[20] Weber T, Blin K, Duddela S, Krug D, Kim HU, Bruccoleri R, Lee SY, Fischbach MA, Müller R, Wohlleben W, Breitling R, Takano E, Medema MH 2015 antiSMASH 3.0—a comprehensive resource for the genome mining of biosynthetic gene clusters. Nucleic Acids Res 43:W237–W243. doi:10.1093/nar/gkv437. 25948579 PMC4489286

[21] Ziemert N, Podell S, Penn K, Badger JH, Allen E, Jensen PR 2012 The natural product domain seeker NaPDoS: a phylogeny based bioinformatic tool to classify secondary metabolite gene diversity. PLoS One 7:e34064. doi:10.1371/journal.pone.0034064. 22479523 PMC3315503

